# Achieving precise chip control for high-end manufacturing

**DOI:** 10.1038/s41598-026-43995-7

**Published:** 2026-03-12

**Authors:** Zhengyang Kang, Qing Guo, Zhen Li, Yuyang He, Yuhao Jiang, Lijia Liu

**Affiliations:** 1 School of Mechanical and Power Engineering, Nanjing Tech University, Nanjing, 211800 Jiangsu China; 2 School of Public Administration, Jiangsu Open University, Nanjing, 211800 Jiangsu China; 3 School of Mechanical Engineering, Jiangsu University, Zhenjiang, 212000 Jiangsu China

**Keywords:** Cutting, Chip control, AISI 316L, Laser ablation, Micro structure, Engineering, Mechanical engineering

## Abstract

In high-end manufacturing, where precision and automation are strictly required, chip control presents an incredibly daunting challenge during the machining process. In this paper, a novel chip control method called grooves induced chip-breaking (GICB) is comprehensively investigated. The core concept of GICB is to achieve spontaneous chip-breaking through the pre-processed micro grooves (PPMG) on the workpiece surface. To understand the underlying chip-breaking mechanisms, PPMG with identical geometric shapes are fabricated on the surface of a 316 L stainless steel workpiece using laser ablation. A cutting experiment is then conducted, where the cutting depth (*d*) and feed rate (*f*) are varied. Experimental observations reveal that, apart from enabling controllable chip-breaking, the GICB method can also enhance the quality of the machined surface. Specifically, the surface roughness (Ra) is reduced by up to 26.6%. Further in-depth analyses indicate that the primary cause of this improvement is the reduced fluctuation of the thrust force. With the assistance of PPMG, the chips produced under controlled periodic fracture exhibit similar lengths and curvatures. Benefiting from that, chip agglomeration and entanglement are effectively avoided. In addition, the fluctuation of cutting force is greatly reduced, which in turn improves the machined surface quality. The practical significance of this study is twofold. Firstly, a high-performance chip control method with significant potential applications is systematically explored. Secondly, for the first time, the crucial relationship between chip control and machined surface quality is experimentally verified.

## Introduction

**Fig. 1:**
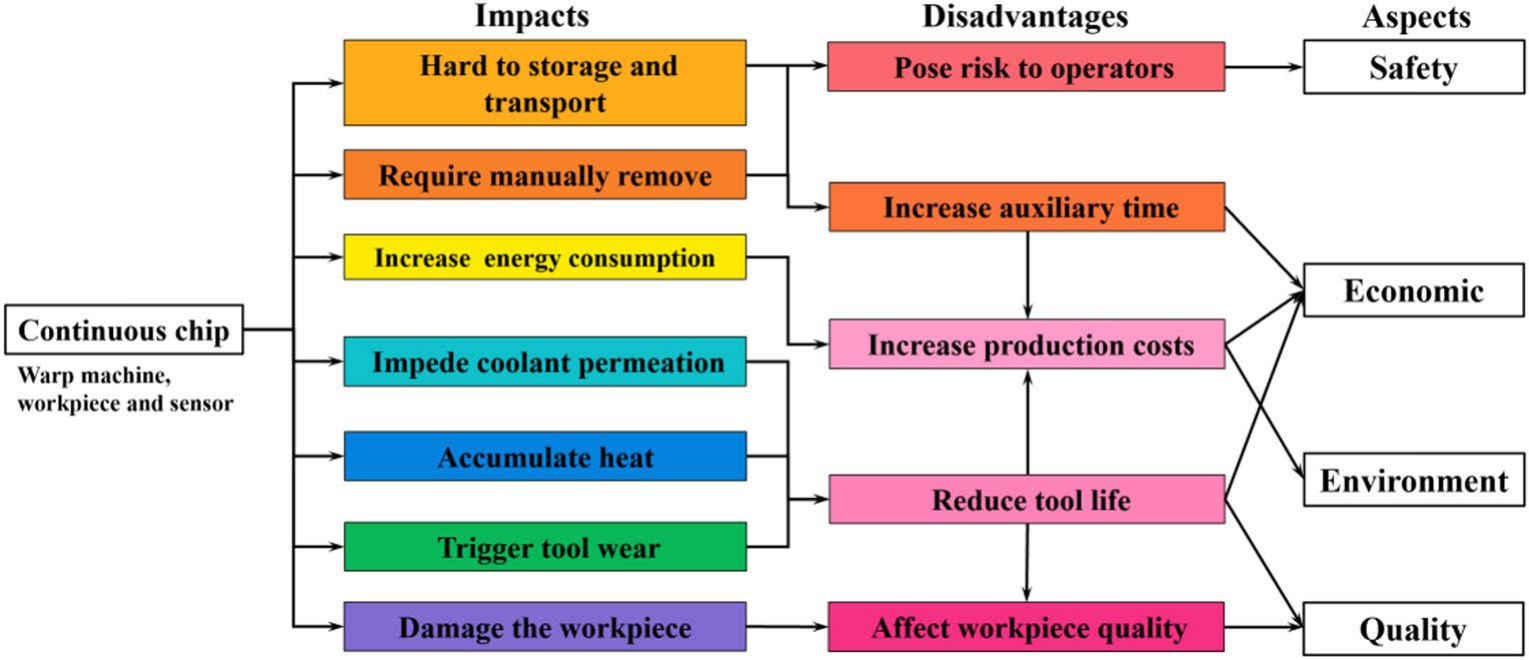
The implications of sub-optimal chip-breaking.

Although metal cutting technologies have witnessed remarkable advancements over the past few decades, achieving controllable chip-breaking remains an arduous task in numerous scenarios within high-end manufacturing^1–3^. In the context of automated machine tools, continuous chips stand as one of the primary contributors to unplanned interruptions. This phenomenon effectively nullifies the efforts and investments channeled into the establishment and operation of smart factories^4–6^. Figure 1 summarizes the impacts of non-ideal chip-breaking on four aspects, including impeding coolant permeation^7^, damaging the machined surface, increasing energy consumption and labor intensity, etc. Indeed, when the entangled chip is ejected with substantial kinetic energy, it can exert a notable impact on the spindle unit, fixture, and the sensors of the machine tool. In contrast, an ideal chip morphology, such as a spiral or “C” shape with a certain length (≤ 50 mm), can avert these risks and confer numerous advantages^8^. Zebala et al.^9^ utilized the high-pressure cooling (HPC) to break the chip in turning of Ti6Al4V. The experimental results indicated that the corrected chip-breaking could reduce power consumption by 0.5 kW, i.e., a reduction of CO_2_ emissions − 734 kg per year-can be optimistically estimated. Because of its importance of safety, economic, environment and quality, chip control has drawn attentions in both fields of academic research and tool manufacturing^7^.

What is the key to achieving chip-breaking? To respond to this query, a great number of researchers made efforts to carry out theoretical studies. In the criterion put forward by Nakayama^10^, the maximum strain of chip, *ε*, exceeds the endurance tension of chip material, *ε*_*B*_, was regarded as the prerequisite of chip-breaking. Assume the cross-section of chip is rectangle, this criterion is given by

1 $$\varepsilon {\mathrm{=}}\frac{{{h_{ch}}}}{2}\left( {\frac{1}{{{R_C}}} - \frac{1}{{{R_L}}}} \right) \geqslant {\varepsilon _B}$$

where *R*_*C*_ is chip initial curvature radius, *R*_*L*_ is blocked chip curvature radius, *h*_*ch*_ is chip thickness.

Equation (1) reveals four potential strategies for chip-breaking:

1. Increasing the chip thickness *h*_*ch*_;
2. Raising the maximum strain of the chip *ε*;
3. Reducing the initial curvature radius of the chip *R*_*C*_;
4. Lowering the endurance tension of the chip material *ε*_*B*_.

Previous chip-breaking methods can be categorized within these approaches. For example, adjusting cutting parameters corresponds to Approach 1, inducing low-frequency vibration or using pressurized coolants aligns with Approach 2, and applying various on-tool chip-breakers pertains to Approach 3. Nevertheless, there is scarce relevant research on Approach 4. One of the rare instances is that the addition of second-phase particles (such as Al₂Cu, Al₆Mn, Si, etc.) to the workpiece material makes the chip more prone to breakage, as reported in^11^. Xing et al.^15,16^ and Arulkirubakaran et al.^17^ claimed that the reduced friction force will lead to more curved chip. 

No single chip control technology can be considered a panacea. For instance, increasing the feed rate and cutting depths can have an impact on the machined surface quality, tool lifespan, and power consumption^7^. Moreover, advanced on-tool chip-breakers are typically designed specifically for certain cutting conditions, which complicates tool selection and management^4,12^. Enhancing the cooling system is effective in reducing cutting force and tool temperature. However, its chip-breaking performance is satisfactory only when the coolant pressure reaches a threshold value, such as 20 MPa^13,14^. One drawback of low-frequency vibration is the degradation of the machined surface quality^18^. Due to these limitations, the existing technologies are still inadequate to address the problem of continuous chips, particularly for some hard-to-machine materials^7^.

To attain high-performance chip control, the current study devised a novel chip-breaking approach designated as GICB. In contrast to conventional chip-breaking methods, which typically concentrate on modifying cutting conditions, the core concept of the GICB method is to directly manipulate the chip’s strength through one or multiple PPMG on the workpiece surface. To mitigate potential negative impacts on cutting continuity, the depth and width of the PPMG were constrained to be less than one hundred microns. Consequently, laser ablation technology was utilized, and the efficacy and characteristics of the GICB method were experimentally explored on a CNC lathe during the turning of AISI 316 L.

## Experimental details

### Fabrication of workpiece

**Fig. 2:**
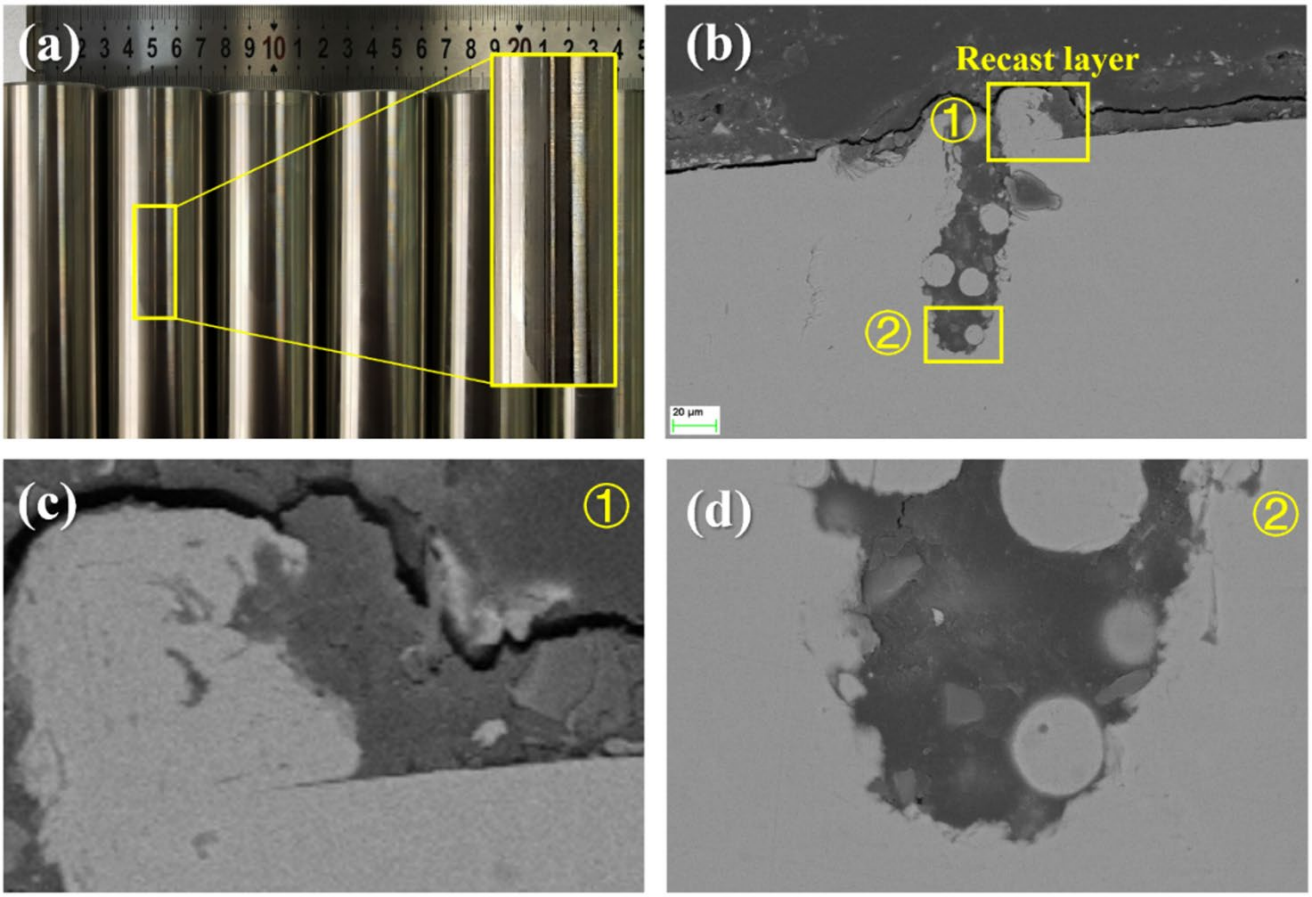
**a** The pre-processed workpieces and **b**–**d** SEM observations of PPMG.

The cylindrical AISI 316 L workpieces were initially machined to the corresponding dimensions: a diameter of (40 ± 0.01 mm), a length of 80 mm, and a surface roughness of Ra 3.7 μm, as shown in Fig. 2a. Subsequently, the workpieces were processed using a fiber laser integrated with a galvo scanning system. For the sake of simplifying the analyses, all the PPMGs were extended along the same axial direction of the workpiece and had identical geometries. The length of the PPMGs was 60 mm. The laser ablation parameters remained constant, with an output laser power of 18 W, a scanning speed of 250 mm/min, a laser pulse frequency of 20 kHz, and a repetition count of 20 times.

Based on the cross-sectional observations presented in Fig. 2b, it can be seen that the width and depth of PPMG were approximately 30 μm and 100 μm, respectively. In addition to the void volume, laser ablation also generated a recast layer on both side of PPMG. From the local enlarged image (Fig. 2c), a gap can be observed in the boundary between the recast layer and the base material. Moreover, as shown in Fig. 2d, the smooth bottom of the PPMG indicated that the actual depth of material damage was equivalent to the depth of the PPMG.

### Cutting experiments

**Fig. 3:**
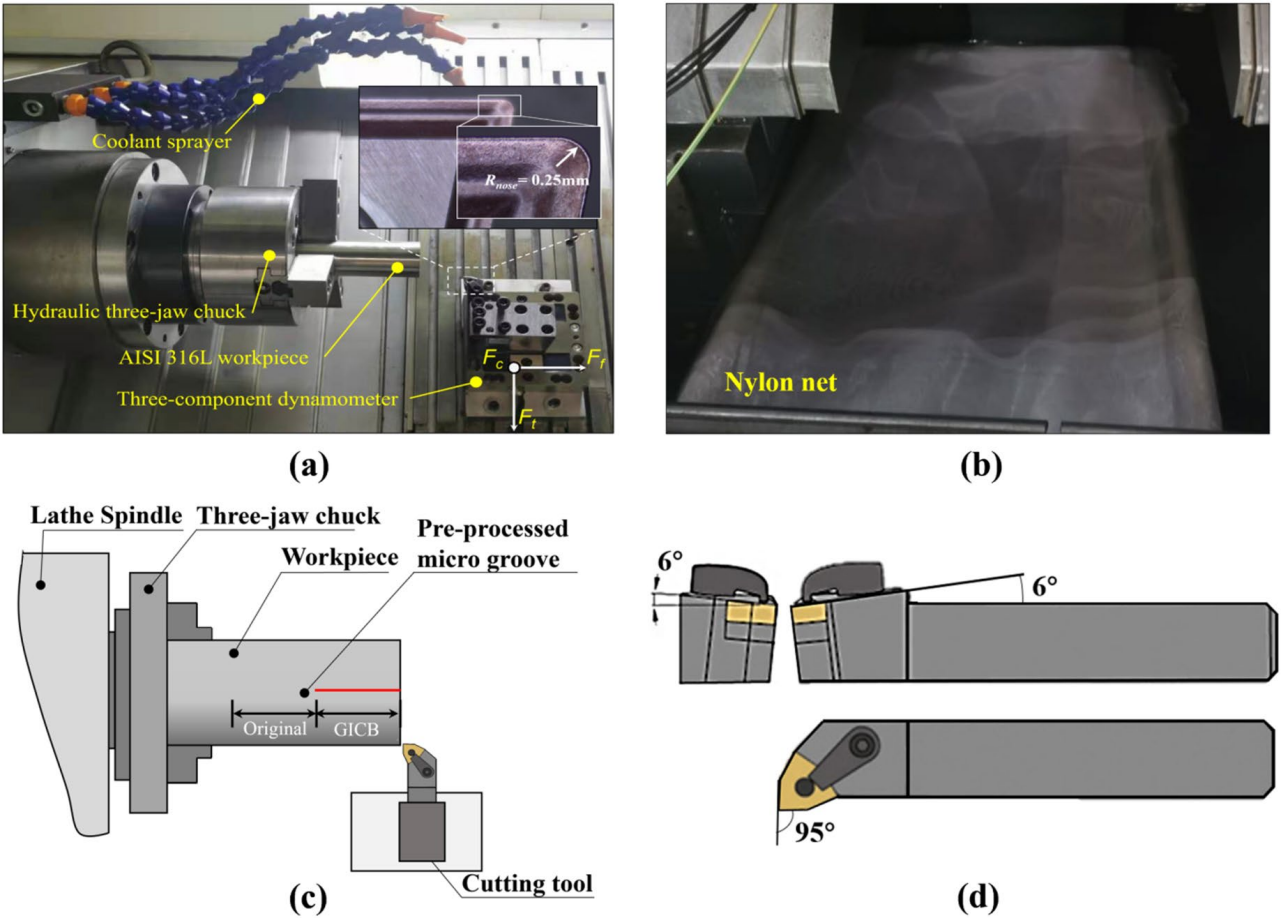
Experimental setup. **a** CNC turning, **b** chip receiver, **c** principle of sectioning, **d** cutting tool.

As shown in Fig. 3a, the cutting experiment was carried out on a double-spindle CNC lathe. A set of force sensors (LH—SZ-05, Shanghai Liheng Sensor Technology) was mounted between the lathe carriage and tool carrier. These sensors were used to measure the triaxial cutting forces. An analog signal acquisition module (USB 3200, Art Technology) with a sampling rate of 50 KS/s was used to acquire the cutting-force signals. These signals were then subsequently processed and converted into graphs for evaluating the effectiveness of the GICB method. The coolant, a phenol-water mixture with a mixing ratio of 1:10, was dispersedly supplied by three low-pressure sprayers, creating a wet-cutting condition. Under such circumstances, the influence of the coolant on chip formation could be ignored.

The workpiece was fixed on a hydraulic three-jaw chuck and its extension length was 50 mm. To minimize the repeat position error of workpieces, the total length of cut (40 mm) was equally divided into original section and GICB section (Fig. 3c). During the follow-up cutting process, these two sections were machined successively with the same cutting parameters, while their surface structures were different. The chip morphologies could be changed evidently in the GICB section because of the intervention of PPMGs. During the cutting, the feed motion paused as soon as the tool reached the boundary of these two sections. During the pause interval, chips were collected using a self-fabricated chip receiver (Fig. 3b). The receiver consisted of a plastic frame covered with a nylon net. This design was intended to minimize unnatural chip-breaking as much as possible.

The cutting tools employed were commercially available uncoated WC/Co cemented carbide (YG6) inserts, which were mounted on a left-hand tool holder. The measured geometric parameters of the tool were as follows: rake angle, *γ*_0_ = 0°, cutting edge inclination angle *λ*_*s*_ = 6°, and major cutting-edge angle, *κ*_r_ = 95°, as depicted in Fig. 3d. The radius of the tool-cutting edge was 40 microns, and the radius of the tool nose was 250 microns. The cutting experiments were carried out within a range of cutting parameters, with *v*_*c*_ = 100 m/min, undeformed chip thickness, *d* = 0.1–0.5 mm, and feed rate, *f* = 0.1–0.5 mm/rev. The detailed experimental parameters are presented in in Table 1.

**Table 1:** Detailed experimental parameters.

Workpiece material	1 PPMG	6 PPMGs
	AISI 316 L	
Coolant	Provided	
Cutting tools	Uncoated, YG6	
Micro groove’s dimension (µm)	30 in width, 100 in depth	
Length of cut (*L*, mm)	20	
Cutting speed (*v*_*c*_, m/min)	100	20
Feed rate (*f*, mm/rev)	0.l, 0.2, 0.3, 0.4, 0.5	0.05
Undeformed chip thickness (*d*, mm)	0.l, 0.2, 0.3, 0.4, 0.5	0.2

The machined surfaces were characterized by a separated type surface roughness tester (Sivaco SF-210).

## Results and discussions

### Characterization of chip morphology

**Fig. 4:**
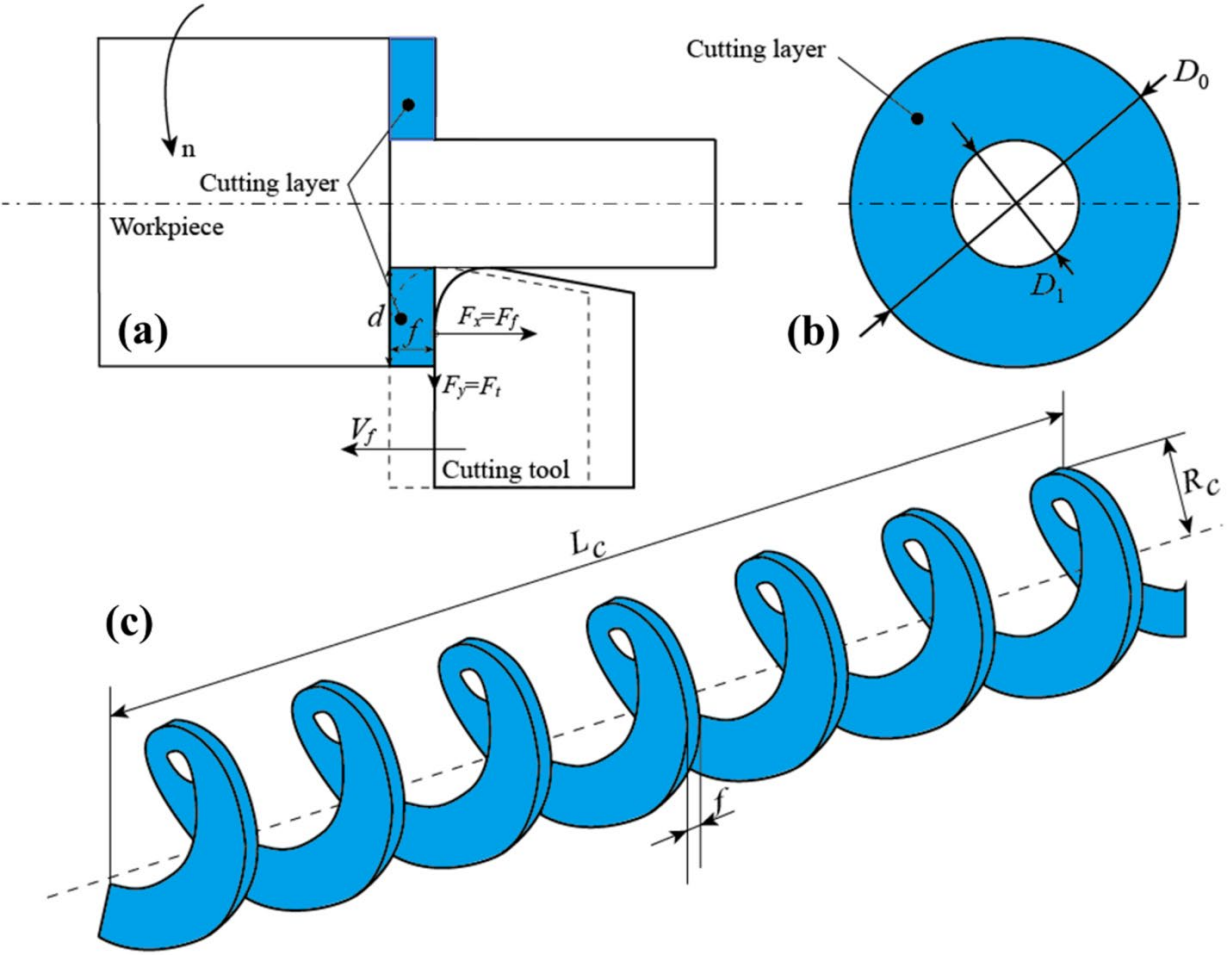
Schematic of chip formation. **a** Cutting layer and its, **b** side view; **c** chip morphology.

In the GICB method, the PPMG only exert an effect when they intersect with the cutting trajectory of the tools. Thus, it is essential to re-examine the material removal process from a new perspective. In the schematic diagram of turning process presented in Fig. 4a, the colored area represents the cross-section of the cutting layer being removed in each rotation of the workpiece. To simplify the analysis, it is assumed that the volume of cutting layer is solely related to the undeformed chip thickness, *d*, workpiece diameter, *D*_*0*_, and feed rate, *f*. Additionally, the tool edge direction angle, *κ*_*r*_, is approximately 90^°^. The removed material (the cutting layer) can be approximately regarded as an annular region in the side-view of the workpiece, as depicted in Fig. 4b. Consequently, the mass of chip, *M*_*c*_, can be estimated as follows:

2 $${M_c}=0.5\pi nfd\rho \left( {{D_0}+{D_1}} \right)$$

where *n* is the workpiece’s rotational number in producing the chip, *ρ* is the density of work material, *D*_0_ is the diameter of original workpiece, and *D*_1_ is the diameter of machined workpiece.

In the present study, the chip morphology was characterized in terms of the chip length, *L*_*c*_, and radius of chip curvature, *R*_*c*_ (as shown in Fig. 4c).

### Experimental results

#### Effects on chip morphology, *f* = 0.1–0.5 mm/r

The workpieces were initially machined with a constant undeformed chip thickness, *d* = 0.2 mm, and cutting speed, *v*_*c*_ = 100 m/min. The feed rate, *f*, was increased from 0.1 to 0.5 mm/r. The morphologies of conventional (continuous, shown in the left-hand figures) and GICB chips (segmented, shown in the right-hand figures) are compared in Table 2. Since these two types of chips were generated successively during a single feed operation, the effectiveness of the GICB method can be reliably evaluated by observing the changes in chip morphologies.

Firstly, all the conventional chips were winding in a disorderly manner, demonstrating a self-tangling tendency. Among them, the chips produced at *f* = 0.4 and 0.5 mm/r had more regular shapes. This is because the chip thickness is positively correlated with the feed rate. These chips had greater strength compared to those produced at *f* = 0.1–0.3 mm/r.

In contrast, for the GICB chips presented in the table column, the most remarkable characteristic was their similar chip length, *L*_*c*_, and radius of chip curvature, *R*_*c*_. This uniformity was more pronounced in the cases of *f* = 0.3–0.5 mm/r, indicating that chip-breaking occurred periodically in the GICB method. It can be further inferred that the PPMG only exert an effect when the cutting trajectory of the tools intersects with them. Therefore, the impact of PPMGs on the overall cutting process is likely to be rather limited, unlike the significant influence of intermittent cutting on cutting force and tool life in the general sense.

**Table 2:**
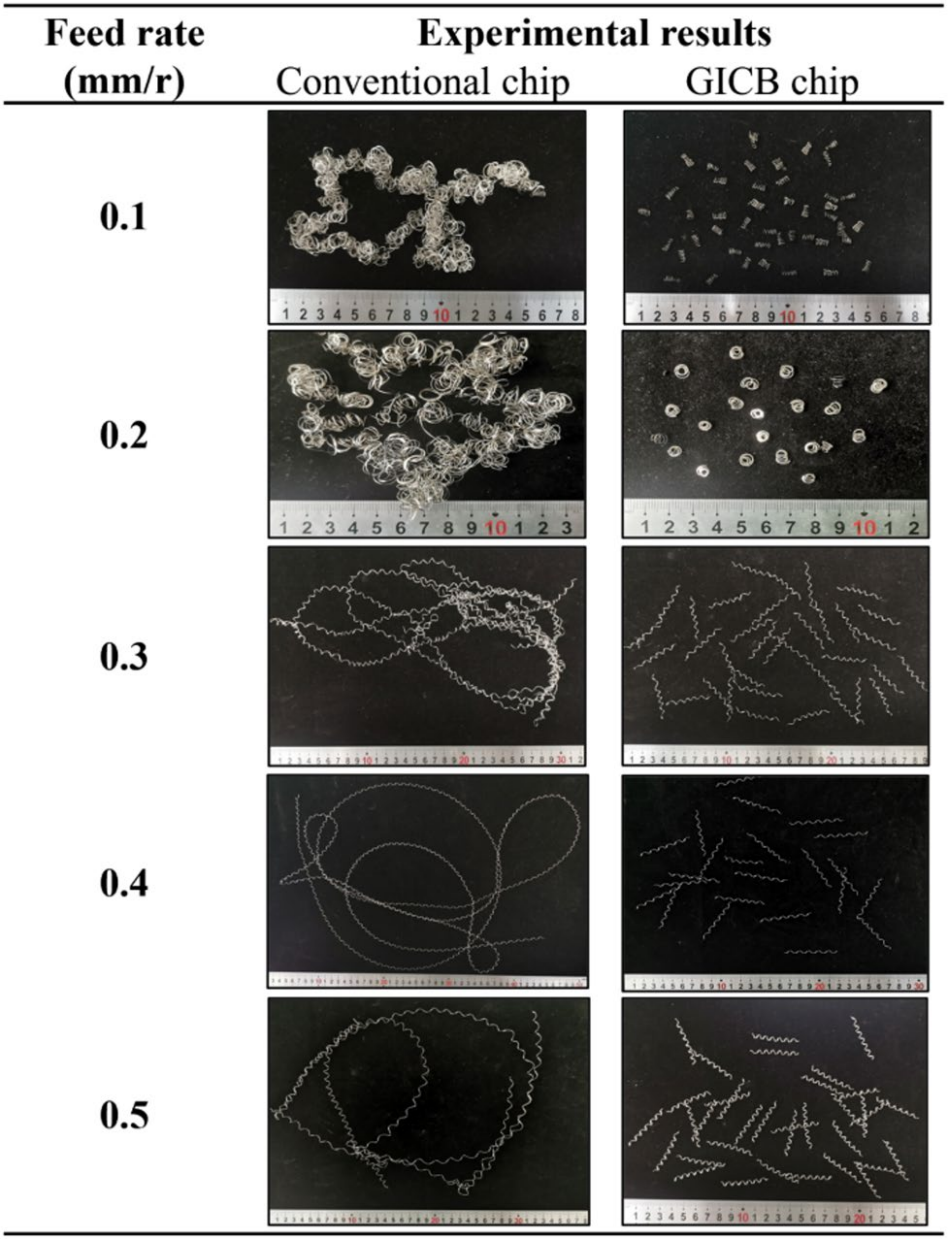
Experimental results at *f* = 0.1–0.5 mm/r, *d* = 0.2 mm, *v*_*c*_ = 100 m/min.

#### Effects on chip morphology, d = 0.1–0.5 mm

In this section, the workpieces were machined at a constant feed rate of *f* = 0.1 mm/r and a cutting speed of *v*_*c*_ = 100 m/min, while the undeformed chip thickness, *d*, was increased from 0.1 to 0.5 mm.

Table 3 compares the morphologies of conventional chips (continuous, presented in the left-hand figures) and GICB chips (segmented, presented in the right-hand figures). As in the previous cases, due to the workpiece material and the selection of non-ideal tools, the conventional chips were not effectively broken. These observations are in line with the common experience that achieving stable and reliable chip-breaking is often extremely challenging, even when modifications to cutting parameters are allowed. In contrast, with the aid of the PPMG, the cutting process in the GICB-based machining generated segmented chips with similar lengths and curvatures. The advantages of periodic chip-breaking will be further explored in the subsequent sections.

**Table 3:**
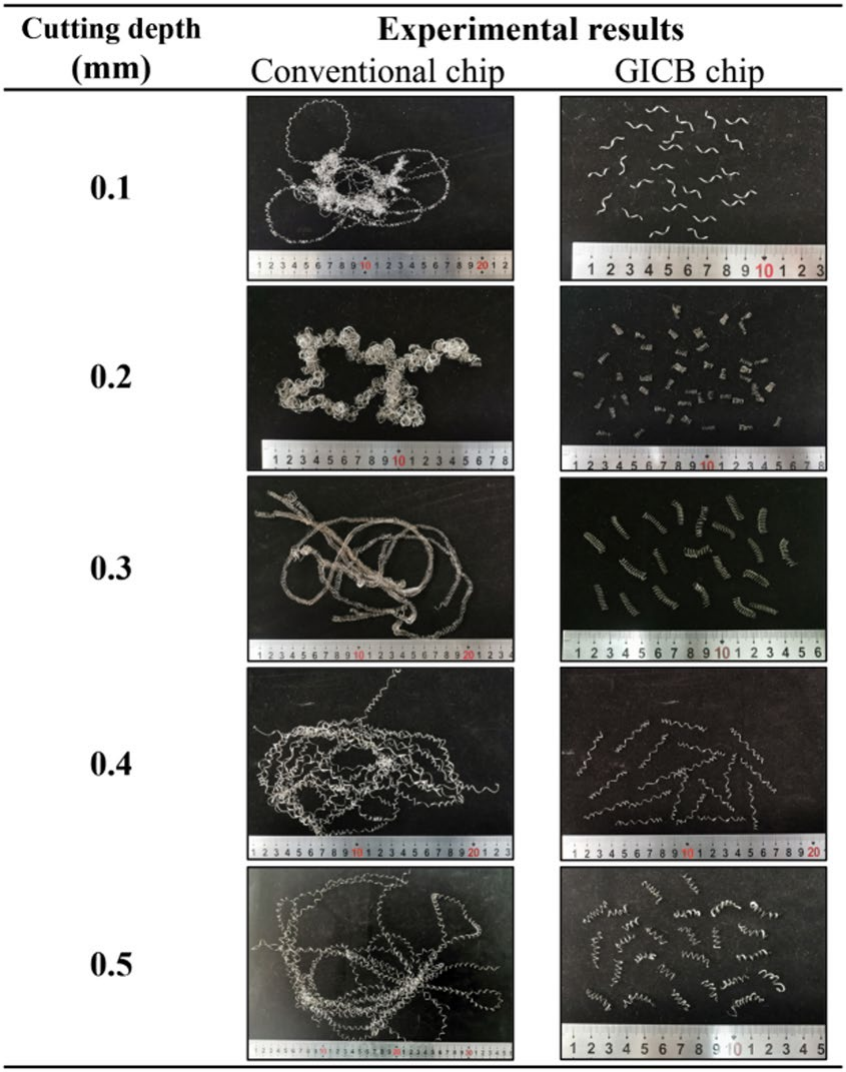
Experimental results at *d* = 0.1–0.5 mm, *f* = 0.1 mm/r, *v*_*c*_ = 100 m/min.

#### Effects on machined surface quality

In present study, with considering the quality of machined surface is of great importance, the depth of micro-grooves was intentionally designed much smaller than the undeformed chip thickness in most of test cases (*d* > 0.1). Therefore, no residual laser-ablated micro-grooves can be observed directly on the machined surface.

**Fig. 5:**
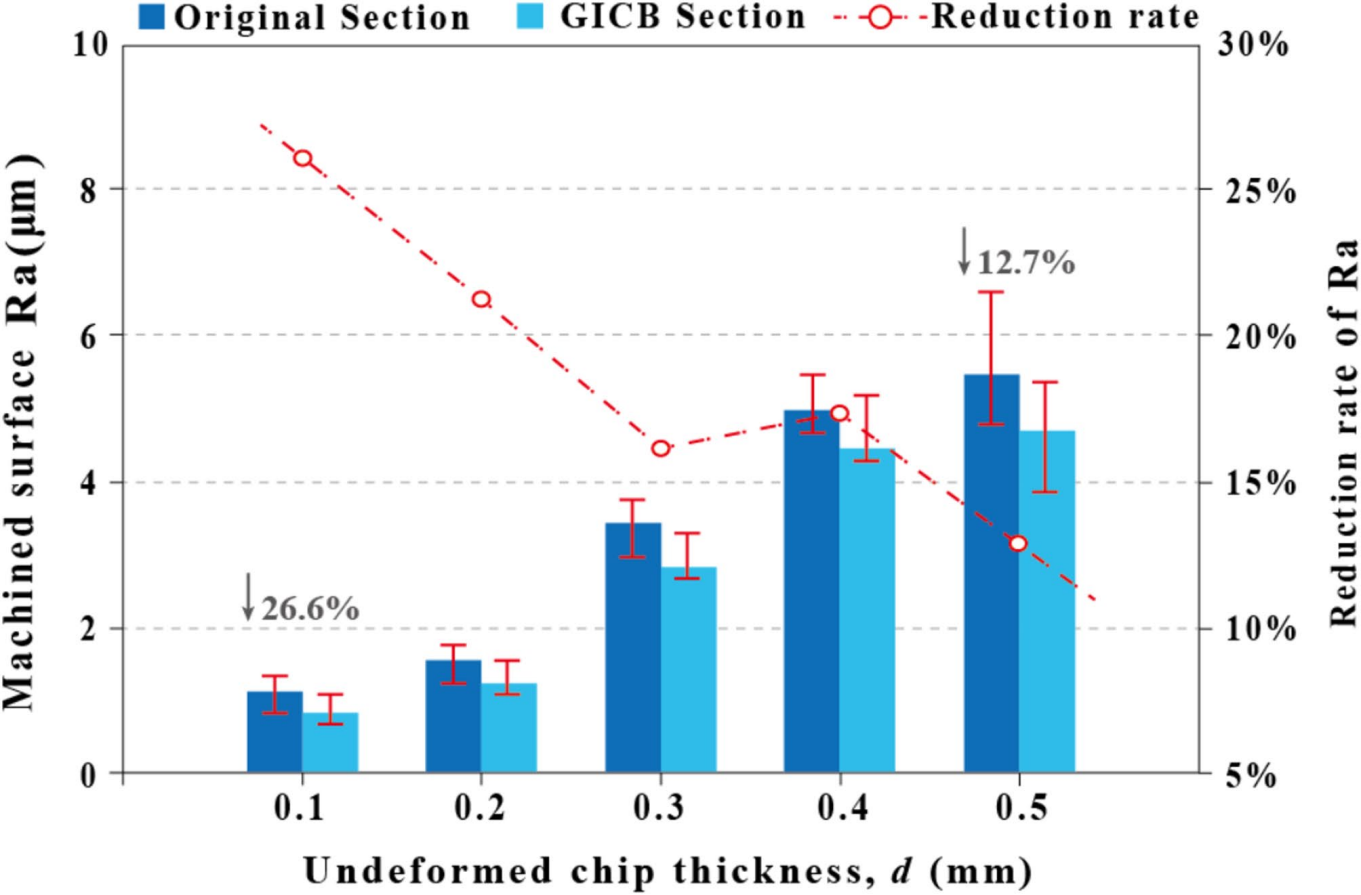
Effects on the surface roughness, Ra, of machined surfaces, *f* = 0.1 mm/r, *d* = 0.1–0.5 mm, *v*_*c*_ = 100 m/min.

Figure 5 compares the *Ra* values of the original and GICB sections. All the machined workpieces exhibited the same pattern: the *Ra* values in the GICB section were lower than those in the original section. Given that these two sections were machined during a single feed under the same cutting conditions, the reduction in *Ra* can be attributed to the beneficial effect of the GICB method.

Overall, the reduction rate of *Ra* decreased as the value of *d* increased. The highest and lowest reduction rates of *Ra* were 26.6% and 12.7%, respectively, occurring in the cases where *d =* 0.1 mm and *d =* 0.5 mm. However, the latter case had the largest decrease in the absolute value of *Ra*. With the increase of the depth of cut, the reduction rate of *Ra* shown a decreasing trend, indicating that the improvement effect of PPMG on the machined surface is more significant within the finishing range.

As shown in Table 3, the original chips were entangled and would frequently collide with the machined workpiece surface, causing damage. In contrast, the well controlled chips did not collide with the finished surface. It could be the main reasons for the observed reduction in surface roughness.

To the best of our knowledge, this study is the first to achieve effective chip control using prefabricated micro-grooves. Accordingly, single-pass cutting was adopted in the experiments to minimize variables. In practical high-end manufacturing, single-pass cutting is widely employed in the final finishing stage of workpieces. Furthermore, uncontrolled chips during finishing represent a critical challenge, as they tend to scratch and damage the pre‑finished smooth surfaces.

For scenarios involving multiple passes, the micro-groove is removed after the first cutting pass and thus can no longer facilitate chip breaking in subsequent passes. In this case, it is only necessary to ensure that the micro-groove is completely removed from the workpiece surface during the final pass; otherwise, the residual micro-groove will impair the surface integrity of the workpiece. This requirement can be easily satisfied based on the present research results.

#### Effects on cutting forces

**Fig. 6:**
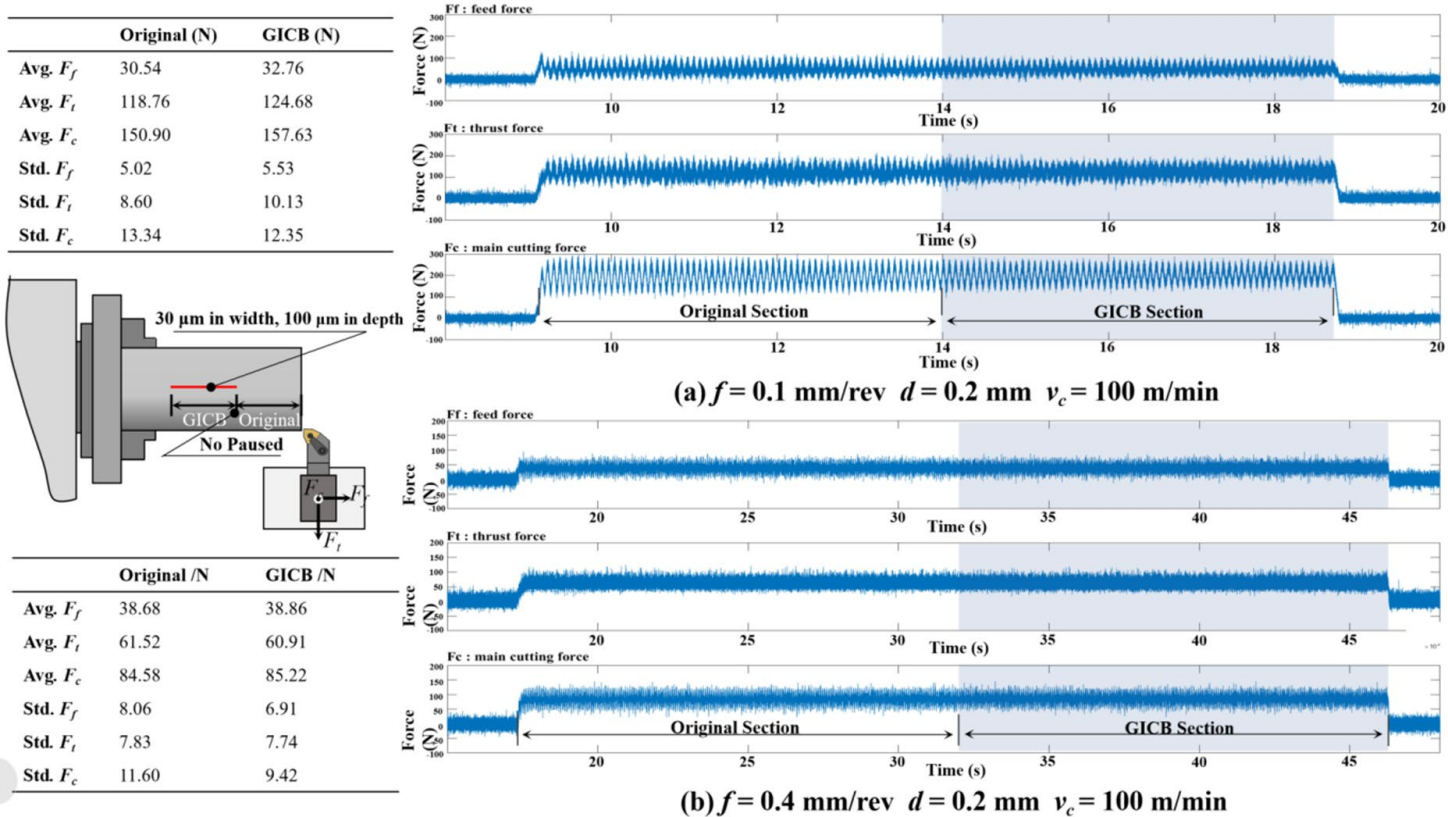
Effects on cutting forces with **a** *f* = 0.1 mm/rev and **b** *f* = 0.4 mm/rev.

Figure 6 illustrates the effects of the PPMG on the cutting forces in time domain. For a more in-depth analysis, the average amplitude (Avg.) and standard deviation (Std.) were utilized to assess the cutting forces and cutting stability, respectively.The original and GICB sections were machined successively during a single feed operation under the same cutting parameters: *f* = 0.1 and 0.4 mm/rev, *d* = 0.2 mm, *v*_*c*_ = 100 m/min. A slight increase can be observed in both the Avg. values and the Std. values of the three force components in the GICB section.

Overall, the PPMG did not exert significant adverse effects on cutting forces, which indicated that in the present study, the micro-grooves with a depth smaller than the depth of cut and hundred micrometers in width exert no significant influence on the cutting process. The following discussion will focus on the beneficial effects of effective chip control on the cutting process.

### Discussions

#### Effectiveness of GICB method

Based on the experimental results presented in Tables 2 and 3, the chip-breaking capabilities of the selected inserts were inadequate under all the tested conditions. Despite the fact that the depth of the PPMGs were merely around 100 microns, their effectiveness persisted as the feed rate or the undeformed chip thickness increased. This covered most of the frequently-used parameters in semi-finishing and finishing operations. Thus, from the analyses of chip morphologies, it can be concluded that the new method is applicable for solving the chip-related issues in the turning of AISI 316 L. Moreover, as the continuous chips were steadily and periodically broken by the PPMG, the morphological uncertainties of conventional continuous chips, such as distortion, self-tangling, and non-uniformity, were effectively eliminated. This phenomenon was more pronounced in the cases where *f* = 0.1–0.3 mm/r since the chips were softer under these conditions.

Conventional methods are unable to achieve periodical chip-breaking due to the complexity and variability inherent in the metal-cutting process. In contrast, the GICB method provides a means to minimize the interference of continuous chips as much as possible. In other words, taking GICB as a benchmark, it is feasible to establish a new system for comprehensively evaluating the applied chip-control measures. During the cutting process, the thrust force is always perpendicular to the workpiece surface. Consequently, the reduction in the standard deviation (Std.) values of the thrust force, *F*_*t*_, monitored in the previous section, can affect the quality of the machined surface. In this study, the roughness, *Ra*, was used to characterize the quality of the machined surface.

#### Amendment of chip-mass estimation

The cutting experiments described above were based on two fundamental conditions:

1. There was only a single PPMG extending along the axial direction of the workpiece.
2. All workpieces had the same diameter, and the rotation speed of the lathe spindle remained constant.

Given these preconditions, chip-breaking induced by the PPMG occurred with each revolution of the workpiece. Thus, the mass of the segmented GICB chip, *M*_*c−GICB*_, can be estimated by

3 $${M_{c{\mathrm{-GICB}}}}=\pi fd\rho \left( {{D_0} - d} \right)$$

**Fig. 7:**
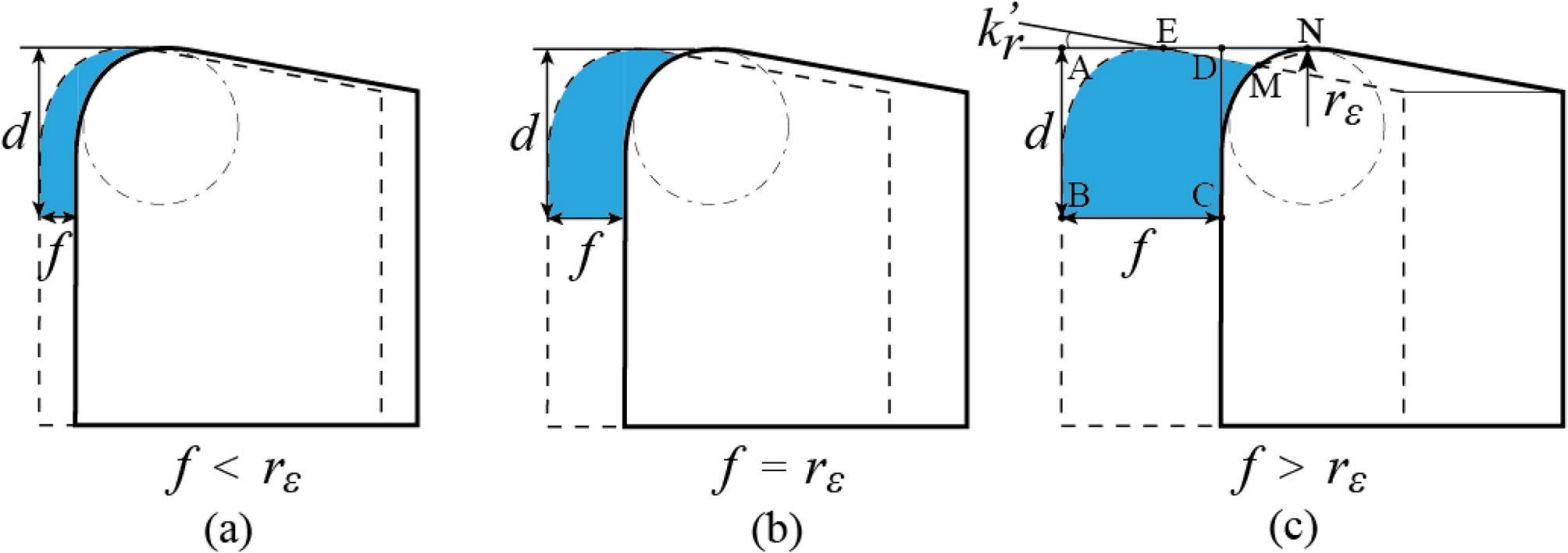
Amendment of uncut region. **a** *f* < *r*_*ε*_, **b** *f* = *r*_*ε*_, **c** *f* > *r*_*ε*_.

If there was only one variable, either *f* or *d*, Eq. 3 represents a linear function. As depicted in Fig. 7, the estimated *M*_*c−GICB*_ (from Eq. 3) qualitatively aligns with the experimental results. However, its prediction accuracy deteriorates as the feed rate *f* increases. Thus, Eq. 3 can be modified by taking into account the tool angles.

During the continuous turning process, the main (solid line) and minor cutting edges (dashed line) create an uncut region. The size of this uncut region is mainly determined by *f*. It can be observed that when *f* > *r*_*ε*_, the uncut region, triangle ΔEMN, cannot be ignored. The modified Eq. 3 is given as follows:

4 $${\hat {M}_{c{\mathrm{-GICB}}}}=\pi \rho \left( {{D_0} - d} \right)({S_{\square ABCD}} - {S_{\Delta EMN}})$$

where *S*_□ABCD_ and *S*_ΔEMN_ are the areas of rectangle ABCD and triangle ΔEMN, they are given by Eqs. 5 and 6

5 $${S_{\square ABCD}}=fd$$

6 $${S_{\Delta EMN}}=0.5f\frac{{f\cot {{k^{\prime}}_r}+{r_\varepsilon } - \sqrt {{r_\varepsilon }^{2}+2f{r_\varepsilon }\cot {{k^{\prime}}_r} - {f^2}} }}{{{\mathrm{1+co}}{{\mathrm{t}}^2}{{k^{\prime}}_r}}}$$

combining Eqs. 4, 5 and 6 gives

7 $${\hat {M}_{c{\mathrm{-GICB}}}}=0.5\pi \rho f\left( {{D_0} - d} \right)\left( {2d - \frac{{f\cot {{k^{\prime}}_r}+{r_\varepsilon } - \sqrt {{r_\varepsilon }^{2}+2f{r_\varepsilon }\cot {{k^{\prime}}_r} - {f^2}} }}{{{\mathrm{1+co}}{{\mathrm{t}}^2}{{k^{\prime}}_r}}}} \right)$$

**Fig. 8:**
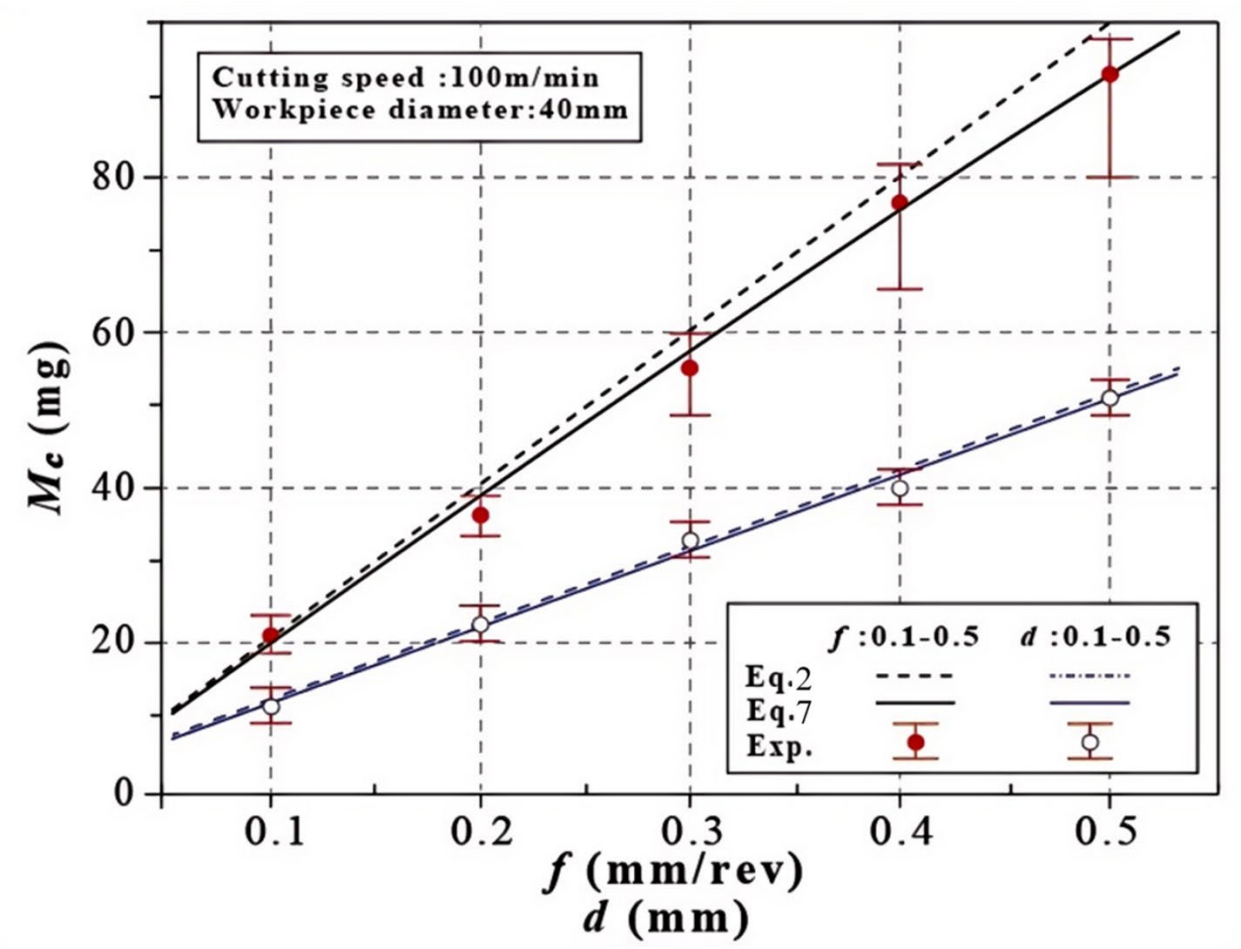
Mass of GICB chip. **a** Effect of *d*. **b** Effect of *f*.

Figure 8 shows the effects of *d* and *f* on *M*_*c−GICB*_. Experimentally, as either *f* or *d* increases, both the upward trend of the average value and the variation of *M*_*c−GICB*_ can be observed. In the case of *f* = 0.1, the predictive lines coincide with the experimental results. However, as *f* increases from 0.1 to 0.4, it becomes evident that Eq. 7 has higher accuracy compared to Eq. 3. The accurate prediction of the segmented chip stems from the fact that the GICB method breaks the chip periodically. This is a highly distinctive feature that differentiates the GICB method from other chip-breaking methods.

#### Analysis of cutting forces in the frequency domain

The time interval *T*_*p*_ during which the tool passes the PPMG can be estimated as follows:

8 $${T_p}={w_f}/{v_c}$$

where *w*_*f*_ is the width of PPMG, *v*_*c*_ is the cutting speed.

**Fig. 9:**
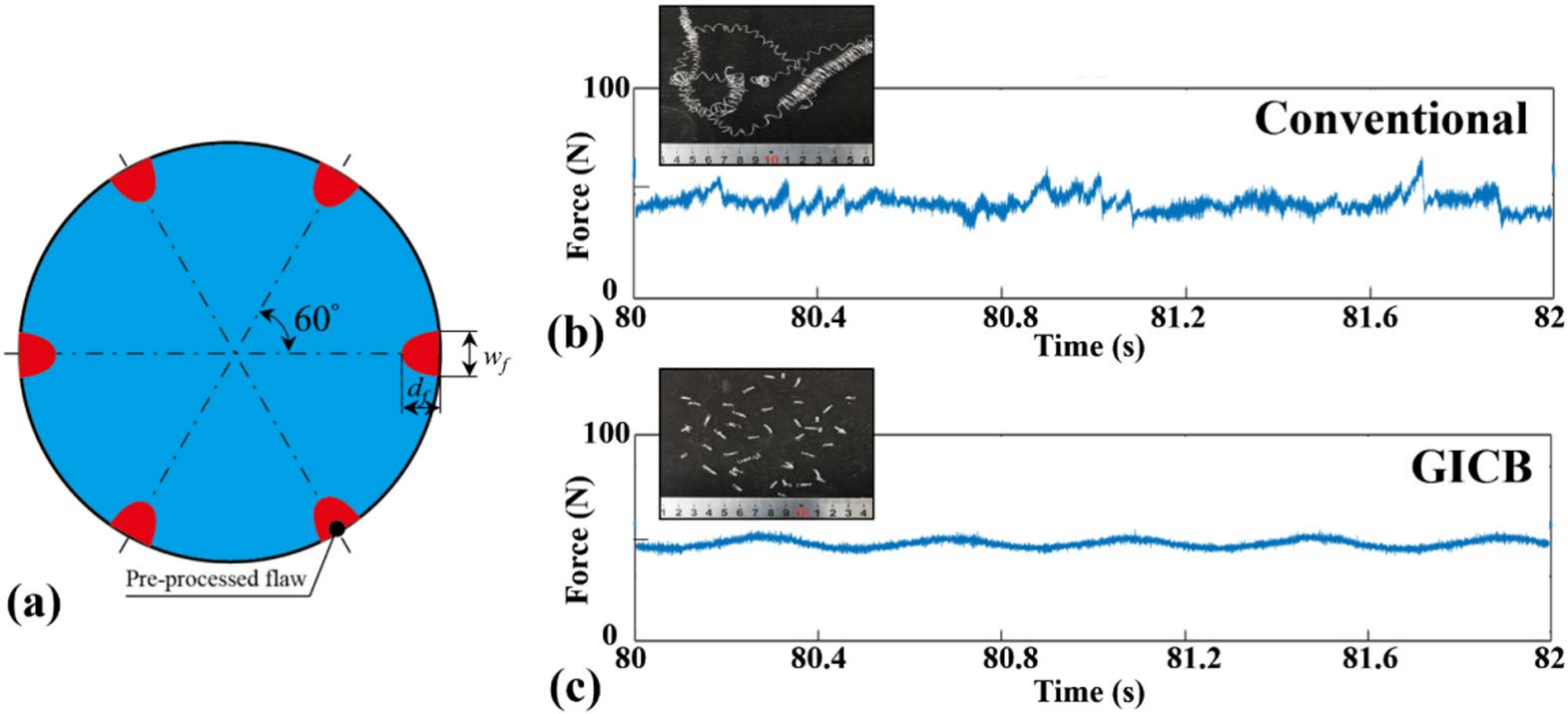
Fluctuation of main cutting forces. **a** Cross-section of the workpiece with six evenly distributed PPMGs. Chip’s morphologies and partial curves of the main cutting force in turning of the **b** conventional and **c** GICB workpiece. *v*_*c*_ = 20 m/min, *f* = 0.05 mm/rev, *d* = 0.2 mm.

For instance, in the case of *w*_*f*_ = 30 μm and *v*_*c*_ = 100 m/min, according to Eq. 8, *T*_*p*_ is merely 18 µs. The fluctuation of cutting force within this time period is extremely difficult to locate and extract from the overall cutting-force signal, which typically persists for tens of seconds. To maximize the value of *T*_*p*_, efforts were made in three aspects: (1) increase the number of PPMGs; (2) reducing the cutting speed; and (3) reducing the feed rate. Eventually, six PPMGs were fabricated, evenly distributed around the circumference of the workpiece (Fig. 9a). The adjusted cutting conditions were *v*_*c*_ = 20 m/min, *d* = 0.2 mm and *f* = 0.05 mm/rev. The precise diameter of the workpiece, was 41.7 mm.

The fluctuation of cutting force serves as an indicator of cutting stability. Initially, we thought that an increased number of PPMGs would lead to more severe cutting-force fluctuations. This was because there were more singularity points along the cutting path. However, as shown by the curves of the main cutting forces, the GICB curve (Fig. 9c) is evidently smoother than the conventional one (Fig. 9b). A plausible explanation for this phenomenon is that the drawbacks of GICB were far outweighed by its advantages in terms of cutting stability. It can be hypothesized that more in-depth mechanisms are at play. Since the chips were periodically broken, most of the random characteristics of the chips were eliminated. Consequently, the PPMGs impart stability rather than causing fluctuations.

Another possible reason is that in the single-groove scheme, every time the tool passes through the groove, the cutting forces temporarily decreases significantly, causing the workpiece to exhibit forced vibration. However, in the six-groove scheme, since the grooves are arranged axisymmetrically about the workpiece axis, the forced vibration is suppressed to a certain extent.

To further analyze the main cutting-force signal in frequency domain, the Fast Fourier Transform (FFT) method is employed. The frequency, *F*_*p*_, at which the cutting tool passes the PPMGs can be calculated as follows:

9 $${{\mathrm{F}}_{\mathrm{p}}}{\mathrm{=}}\frac{{{\mathrm{N}}{{\mathrm{v}}_{\mathrm{c}}}}}{{{\mathrm{\boldsymbol{\uppi}}}{{\mathrm{D}}_{\mathrm{0}}}}}$$

where *N* represents the number of PPMGs. *D*_0_ is the diameter of original workpiece. According to Eq. 9, in case of six PPMGs (*N = 6*), the precise diameter of the workpiece was 41.7 mm (*D*_*0*_ = 41.7), *v*_*c*_ = 20 m/min, the value of *F*_*p*_ is approximately 15.3 Hz.

**Fig. 10:**
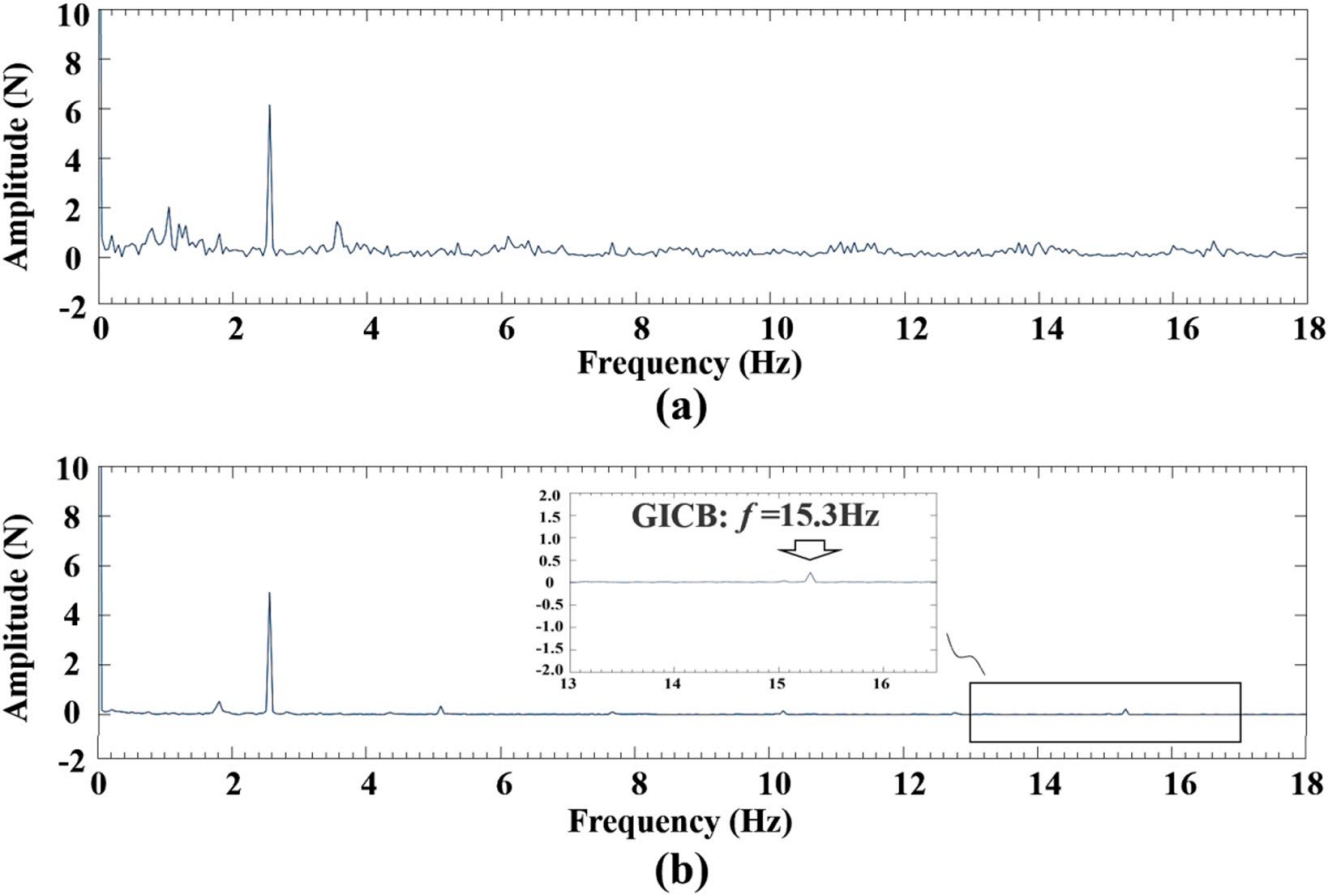
Frequency domain analysis of main cutting forces. **a** The conventional workpiece and **b** GICB workpiece. *v*_*c*_ = 20 m/min, *f* = 0.05 mm/rev, *d* = 0.2 mm.

Figure 10a depicts that the cutting-force spectrum during the turning of a conventional workpiece exhibits numerous scattered peaks. In contrast, in the case of GICB (Fig. 10b), only a few peaks are observable, and one of them is a 15.3 Hz signal that corresponds to the calculated value of *F*_*p*_. However, the amplitude (signal strength) of the 15.3 Hz signal is not markedly higher than that of the other peaks. This indicates that the influence of the PPMGs on turning stability is not prominent, which is consistent with the time-domain analysis presented in Fig. 9c.

Based on the foregoing analysis, it can be ultimately concluded that the uncontrolled continuous chip was the primary source of cutting instability. The random characteristics of the continuous chip, such as distortion, vibration, friction, and fracture, augmented the fluctuation of the cutting force. Therefore, in addition to the proactive and periodic chip-breaking, another significant advantage of the GICB method is that it can remarkably suppress the randomness of the chip. As shown in Fig. 10b, the dynamic component in the cutting-force signal was reduced by approximately 85%.

## Conclusions

In the present study, the groove induced chip breaking (GICB) method is experimentally investigated in the turning of AISI 316 L. The effectiveness of the GICB method has been verified across a wide range of finish-cutting conditions: cutting speeds 20 and 100 m/min, undeformed chip thicknesses from 0.1 to 0.5 mm, and feed rates from 0.1 to 0.5 mm/rev. The relevant conclusions are as follows:

1. The specific dimensions of the pre-processed micro grooves (PPMGs), approximately 30 μm in width and 100 μm in depth, enable effective chip-breaking over a wide range during the turning of AISI 316 L, encompassing most of the finish-cutting conditions. The maximum value of the undeformed chip thickness, 0.5 mm, is five times greater than the depth of the PPMGs. It can be concluded that the GICB method can be applied to address the problem of continuous chips in the turning of AISI 316 L.
2. The periodicity of chip-breaking in the GICB method has been proven in three ways: (1) by observing the chips’ morphologies; (2) by comparing the measured and estimated chip masses; and (3) by analyzing the cutting-force signal in both the time-domain and frequency-domain. Considering the uncut regions generated by the minor cutting edge, the accuracy of chip-mass estimation can be further promoted.
3. The controlled chip-breaking can effectively eliminate the random characteristics of chips. A significantly smoother curve of the main cutting-force signal can be obtained due to the PPMGs on the workpiece surface. The dynamic component in the cutting-force signal is reduced by approximately 85%.

## Data Availability

The data that support the findings of this study are available from the corresponding author upon reasonable request.

## Abbreviations

*d*: Undeformed chip thickness
*v*_*c*_: Cutting speed
*D*_0_: Diameter of original workpiece
*D*_1_: Diameter of machined workpiece
*r*_*β*_: Cutting edge radius
*r*_*ε*_: Tool-nose radius
$k_{r}^{\prime }$: Tool minor cutting edge angle
*M*_*c*_: Mass of chip
*S*_0_: Area of chip’s cross section
*w*_*f*_: Width of pre-processed flaw
*d*_*f*_: Depth of pre-processed flaw
*S*_0_: Area of chip’s cross section
*L*_*c*_: Length of chip
*R*_*c*_: Chip curvature radius
*T*_*p*_: Time of tool passes the pre-processed micro groove
*F*_*p*_: Frequency of tool passes the pre-processed micro grooves
*N*: Number of pre-processed micro grooves
